# Small molecule inhibition of RAS/MAPK signaling ameliorates developmental pathologies of Kabuki Syndrome

**DOI:** 10.1038/s41598-018-28709-y

**Published:** 2018-07-17

**Authors:** I-Chun Tsai, Kelly McKnight, Spencer U. McKinstry, Andrew T. Maynard, Perciliz L. Tan, Christelle Golzio, C. Thomas White, Daniel J. Price, Erica E. Davis, Heather Amrine-Madsen, Nicholas Katsanis

**Affiliations:** 1 Center for Human Disease Modeling, Duke University School of Medicine, Durham, NC 27701 USA; 2 Target Sciences, GlaxoSmithKline, Research Triangle Park, NC 27709 USA; 3 Platform Technology and Science, GlaxoSmithKline, Research Triangle Park, NC 27709 USA

## Abstract

Kabuki Syndrome (KS) is a rare disorder characterized by distinctive facial features, short stature, skeletal abnormalities, and neurodevelopmental deficits. Previously, we showed that loss of function of RAP1A, a RAF1 regulator, can activate the RAS/MAPK pathway and cause KS, an observation recapitulated in other genetic models of the disorder. These data suggested that suppression of this signaling cascade might be of therapeutic benefit for some features of KS. To pursue this possibility, we performed a focused small molecule screen of a series of RAS/MAPK pathway inhibitors, where we tested their ability to rescue disease-relevant phenotypes in a zebrafish model of the most common KS locus, *kmt2d*. Consistent with a pathway-driven screening paradigm, two of 27 compounds showed reproducible rescue of early developmental pathologies. Further analyses showed that one compound, desmethyl-Dabrafenib (dmDf), induced no overt pathologies in zebrafish embryos but could rescue MEK hyperactivation *in vivo* and, concomitantly, structural KS-relevant phenotypes in all KS zebrafish models (*kmt2d*, *kmd6a* and *rap1*). Mass spectrometry quantitation suggested that a 100 nM dose resulted in sub-nanomolar exposure of this inhibitor and was sufficient to rescue both mandibular and neurodevelopmental defects. Crucially, germline *kmt2d* mutants recapitulated the gastrulation movement defects, micrognathia and neurogenesis phenotypes of transient models; treatment with dmDf ameliorated all of them significantly. Taken together, our data reinforce a causal link between MEK hyperactivation and KS and suggest that chemical suppression of BRAF might be of potential clinical utility for some features of this disorder.

## Introduction

Kabuki syndrome (KS) is a developmental disorder characterized by a distinctive set of facial features, short stature, intellectual disability, dermatoglyphic abnormalities, and internal malformations of the cardiac, renal, gastrointestinal, and/or skeletal systems^1–4^. The global prevalence has been estimated at 1:32,000 births^5^. Current treatment options for KS do not exist, with clinical care limited to the management of individual symptoms^6,7^. The lack of a KS-specific treatment has motivated research into the genetic and pathomechanistic bases of the disorder, although the rarity of this syndrome continues to pose commercial and regulatory challenges in the pursuit of novel therapeutic approaches.

Mutations in lysine (K)-specific methyltransferase 2D (*KMT2D*, also known as *MLL2*)^8–11^ and lysine (K)-specific demethylase 6 A (*KDM6A*)^11–13^ are mutated in ~75% and 5% of KS cases, respectively. Subsequent to these discoveries, we reported that mutations in the genes coding for two RAS-related proteins, RAP1A and RAP1B, can also cause KS and the phenotypically-overlapping Hadziselimovic syndrome. Grounded on these observations and the known role of RAP1 in the regulation of RAS/MAPK signaling^14^, we showed in zebrafish embryos that dysfunction of any of *kmt2d*, *kdm6a*, *rap1a*, or *rap1b* yields anatomical developmental defects relevant to the KS phenotype through aberrant hyperactivation of MEK within the RAS pathway^15^. During gastrulation, these phenotypes manifested as convergence and extension (CE) defects; later in development, we also observed cell-cell intercalation pathologies that likely drive mandibular formation defects and, ultimately, micrognathia^15^.

The RAS/MAPK pathway begins with RAS activation, which promotes the activation of RAF protein kinases, including ARAF, BRAF, and/or RAF1 (Supplementary Fig. 1). RAF kinases phosphorylate and activate MEK1 and/or MEK2, which in turn phosphorylate and activate ERK1 and/or ERK2. ERK1/2 is the ultimate effector; its substrates include nuclear components, transcription factors, membrane proteins, and protein kinases that control a multitude of processes such as cell cycle progression, differentiation, and growth^16^.

In humans, the small GTPases RAP1A and RAP1B regulate RAS/MAPK signaling^14^. In some contexts, these proteins act to inhibit the phosphorylation of RAF1, and are thus antagonists of MAPK signal propagation^17^. In other contexts, RAP1A and RAP1B activate BRAF and thus agonize MAPK signaling^18,19^. In KS, we have shown that loss of the RAF1-inhibitory activity of RAP1A or RAP1B is the likely driver of developmental pathologies, not least because we were able to rescue CE-driven phenotypes by suppressing RAF1 genetically in *rap1* mutants. Moreover, we were able to phenocopy this rescue by downregulating MEK signaling by exposing morphant KS embryos to the small molecule tool compound PD184161, a MEK inhibitor^15^.

Together, these results suggested that some of the features found in KS patients overlap mechanistically with the “RASopathies,” a group of disorders caused by germline mutations in genes that encode components or regulators of the RAS/MAPK pathway^20,21^. Although each RASopathy is unique, they all share characteristics with KS, such as craniofacial dysmorphisms; musculoskeletal, cutaneous, and cardiac abnormalities; and neurocognitive impairment^20^. Importantly, this group of disorders has remained too rare to motivate robust *ab initio* drug discovery efforts. However, the RASopathies may benefit from a serendipitous advantage, in that persistent activation of the RAS/MAPK pathway has been reported in several cancers^22^. Most notably, activating mutations in BRAF lead to constitutive activation and phosphorylation of MEK and ERK in the RAS-RAF-MAPK signaling cascade, which are understood to contribute significantly to malignant melanoma, thyroid and colon carcinomas, as well as other cancers^23^. As a consequence, drug discovery efforts have led to the development of clinically approved inhibitors that are now prescribed for these cancers^24^.

The conceptual bridge between the development of small molecule inhibitors for somatic RAS/MAPK activating mutations and their possible utility in germline disorders of this pathway has some experimental support. For example, treatment of a *Raf1* mouse model of Noonan syndrome with a MEK inhibitor ameliorated several key pathologies, including short stature, facial dysmorphologies, and cardiac defects^25^. Similarly, developmental brain abnormalities of a neurofibromatosis type 1 mouse model were extinguished by neonatal administration of a MEK/ERK pathway inhibitor^26^. Some of these therapeutic avenues have now advanced to clinical trials in humans^21^.

Here, we took advantage of the malleability and physiological relevance of our zebrafish KS models to test this hypothesis by screening a focused collection of chemically diverse kinase inhibitors of BRAF, MEK, and ERK for their ability to ameliorate KS deficits. We found several compounds that could rescue phenotypes in KS zebrafish models; however, our most promising result is desmethyl Dabrafenib (dmDf), a soluble BRAF inhibitor that rescues not only the CE defects of KS morphant and mutant zebrafish embryos, but also the craniofacial and neuroanatomical defects of *kmt2d*-, *rap1-* and *kdm6a-*depleted zebrafish larvae, across both transient and stable genetic models. This inhibitor therefore holds promise for treating some of the features of KS with a possible expansion of its utility to other RASopathies.

## Results

### Screening of chemical compounds for potential KS treatments

**Figure 1:**
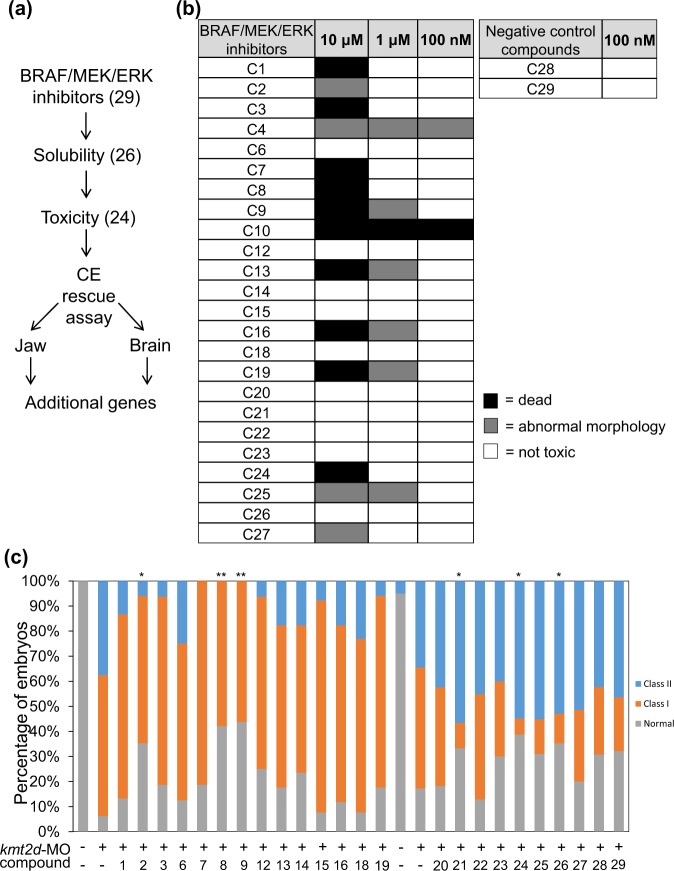
*In vivo* screen using BRAF/MEK/ERK inhibitors. (**a**) Flow chart of the experimental design. We started the screening with 29 compounds (27 active BRAF/MEK/ERK inhibitors, and 2 negative controls). 26 compounds passed the solubility test and 24 of those passed the toxicity test. For the primary screen, 24 compounds were assessed for their ability to rescue the convergent-extension (CE) phenotype of zebrafish *kmt2d* morphants, followed by rescue tests of additional phenotypes (jaw and brain) within the *kmt2d* morphant, or CE of additional genetic KS models. (**b**) Toxicity results. Twenty embryos at the 8-cell stage were incubated in egg water containing 100 nM, 1 μM, and 10 μM of the 26 compounds. After 24 hours, larvae were scored for toxicity. Black bar: death; grey bar: abnormal morphology; white bar: nontoxic. (**c**) Embryos were injected with *kmt2d*-MO at the 2–4 cell stage and soaked in egg water containing 100 nM of each compound at the 8-cell stage. After 24 hours, larvae were scored for CE. Depletion of *kmt2d* resulted in mild (Class I, orange) and severe (Class II, blue) CE deficiency during gastrulation. The efficacy of each compound was determined by a chi-square test in which Class I and Class II were considered together. Note that the compounds were tested in two groups (compounds 1–19 and compounds 20–29), each with its own set of controls. Compound 29, an inactive Braf inhibitor, served as a negative control. *p < 0.05 and **p < 0.01 for compounds that improved the phenotype compared to *kmt2d-*MO alone.

We obtained a series of 27 validated tool BRAF, MEK, and ERK antagonists, as well as two inactive BRAF inhibitor analogs. To guard against possible bias, the tool and negative control compounds were interspersed across the experiment and the zebrafish phenotyping team was blinded to the identity of each compound. Typical of small molecule storage, each of the 29 compounds was dissolved in dimethyl sulfoxide (DMSO). Therefore, we first diluted each in water (100x) and tested its solubility in egg water; three compounds were insoluble and were thus removed from further testing (Fig. 1a). Next, we diluted serially each compound with egg water and tested their toxicity by assaying the percentage of abnormal or dead embryos across three different concentrations (10 μM, 1 μM, 100 nM; Fig. 1b). We found that 22/26 small molecules induced no appreciable pathology at a concentration of 100 nM (Fig. 1b); we therefore selected that dose for subsequent experiments.

We screened this library against our validated transient suppression reagent for *kmt2d*. We injected *kmt2d*-morpholino (MO) into one- to two-cell-stage embryos and soaked them in egg water containing 100 nM of each compound approximately one hour after injection (eight-cell stage). Embryos were allowed to grow to the 8–10 somite stage, at which point they were scored for CE phenotypes using previously established objective qualitative criteria (normal; class I, which have grossly normal morphology, but are shorter than control-injected embryos; or class II, which are shorter and thinner than class I embryos and have poorly developed head, eye, and tail structure, with poor somitic definition and symmetry^27^). Compared to a vehicle control (treatment with DMSO), treatment with compounds 2, 8, 9, 21, 24, and 26 (structures of compounds 2 and 8 given in Supplementary Fig. 2) decreased the proportion of embryos showing CE defects (though note that compounds 21, 24, and 26 appeared to increase the proportion falling into Class II; Fig. 1c). Reassuringly, post hoc analysis of the identity of the compounds revealed that neither of the two negative controls (compounds 28 and 29 (Fig. 1c)) had an appreciable effect.

### Compounds 2 and 8 ameliorate CE and jaw defects of kmt2d, kdm6a and rap1 models

To identify candidates suitable for further preclinical studies, we next asked (1) which of the six compounds that could rescue *kmt2d* MO-induced CE could be reproduced; (2) whether the compounds identified from our screen could also rescue the craniofacial anomalies seen in our KS zebrafish *kmt2d* morphants; and (3) whether these compounds could also rescue the defects in other KS zebrafish models.

**Figure 2:**
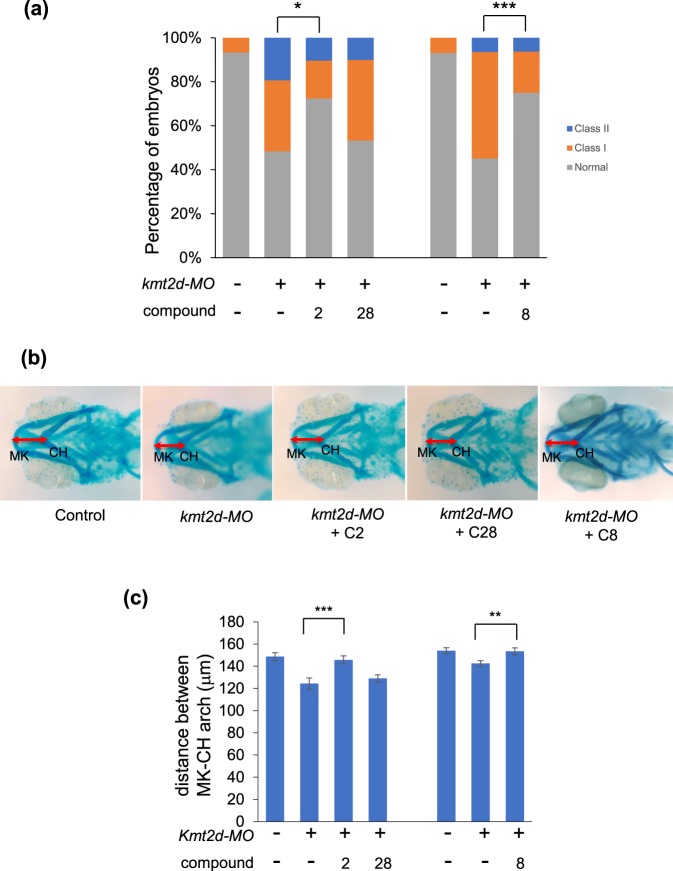
Compounds 2 and 8 ameliorate CE and jaw defects caused by loss of *kmt2d*. (**a**) Compounds 2 and 8, but not 28 (negative control), rescue CE defects of *kmt2d* morphants. (**b**) Alcian blue staining of jaw. Depletion of *kmt2d* leads to a change in jaw layout, reducing the distance between Meckel’s (MK) cartilage and ceratohyal (CH) cartilage (red double arrow). Treatment with compound 2 or 8, but not 28, ameliorates this defect in *kmt2d* morphants. (**c**) Quantitative measurement of the distance between MK and CH cartilages (n > 25). *p < 0.05; **p < 0.01; ***p < 0.001. Error bars show SEM (standard error of the mean).

**Figure 3:**
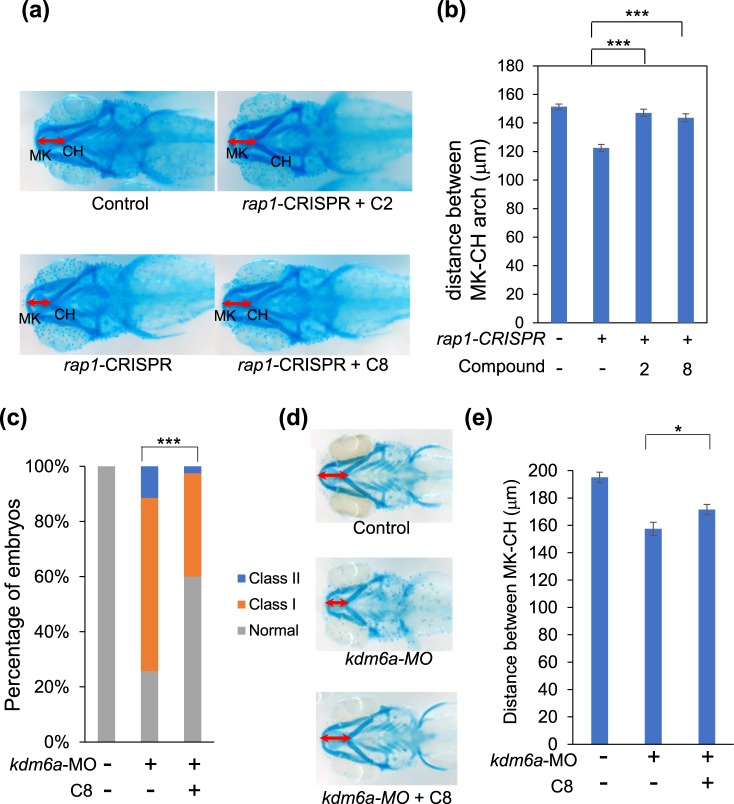
Compound 8 ameliorates the CE and jaw defects caused by loss of *rap1* and *kdm6a*. (**a**) gRNA targeting both zebrafish *rap1a* and *rap1b* were injected into embryos at the 1-cell stage with Cas9 protein. Larvae were incubated with compound 8 (100 nM) at the 8-cell stage and scored for micrognathia. 5 dpf larvae were stained with Alcian blue to assess the layout of the jaw cartilage. In comparison to control embryos (top), *rap1*-CRISPR embryos have smaller jaws. This phenotype can be ameliorated by treatment with compound 2 and 8. (**b**) Quantitative measurement of the distance between MK and CH cartilages. (**c**) Larvae were scored for CE (n > 30 for each concentration). (**d**) 5 dpf embryos were stained with Alcian blue to assess the layout of jaw cartilage. In comparison to control embryos (top), *kdm6a*-MO embryos exhibit smaller jaws. This phenotype can be ameliorated by treatment with compound 8. (**e**) Quantitative measurement of the distance between MK and CH cartilages. *p < 0.05; ***p < 0.001. Error bars show SEM (standard error of the mean).

To test the first criterion, we repeated the CE rescue paradigm four times by two different investigators, each using independent zebrafish adult stocks. We found that compounds 2 and 8 gave consistent and significant rescue of CE in *kmt2d* morphants across all experiments (representative data, Fig. 2a). For the second criterion, we raised larvae in the presence of compound to 5 days post fertilization (dpf), fixed and stained cartilage structures with Alcian blue, and measured the distance between the Meckel’s (MK) and ceratohyal (CH) cartilages, which we have shown previously to be reduced significantly in *kmt2d* morphants and *rap1* mutants and morphants^15^. Consistent with our CE data, and with the expectation that CE defects and micrognathia are both likely driven by aberrant cell intercalation^28,29^, we found that treatment of *kmt2d* morphants with compounds 2 and 8 could rescue significantly the jaw defects as indicated by MK-CH distance (Fig. 2b,c). Next, we asked whether compounds 2 and 8 could ameliorate the jaw pathology of additional KS zebrafish models, including a *kmd6a* morphant (*kmd6a* has been refractory to CRISPR/Cas9 mutagenesis) and a *rap1* F0 CRISPR mutant^15^. For this purpose, we assayed larvae at 5 dpf using the same experimental conditions as for our *kmt2d* morphants. Bathing embryos in either compounds 2 or 8 (Supplementary Fig. 2) restored significantly the jaw length of morphants and mutants to nearly wild type levels (Fig. 3a,b,d,e).

### Exposure of compounds 2 and 8 in zebrafish embryos

Our data suggest that both compounds 2 and 8 offer proof of concept that inhibiting the MEK/ERK pathway may improve features of KS. We therefore wondered about exposure of these molecules in the zebrafish embryos at the dosage that achieved significant rescue (100 nM). For this purpose, we performed mass spectrometry (MS) in embryos treated with each of compounds 2 and 8. Wild-type embryos were treated with 100 nM (the concentration used for the rescue experiments) or 1 µM (as a positive detection threshold given that the intracellular concentration of each compound is unknown at the beginning of the experiment). After five days of treatment, embryos were harvested and subjected to MS analysis (Supplementary Fig. 4a). In addition to compounds 2 and 8, we also assayed the following controls: (1) Compound 29, an inactive BRAF inhibitor, which served as a standard for calibration; (2) Cisapride, which was included to assess the efficiency of homogenization^30^; and (3) Compounds 6 and 22, which are BRAF and ERK inhibitors, respectively, that did not rescue significantly the KS zebrafish models. We observed detectable concentrations of compounds 2, 8, 6, and 22, indicating that all these compounds, including the compounds that failed to rescue KS phenotypes, can be delivered into zebrafish embryos via the bathing method (Supplementary Fig. 4b).

Unmasking the identity of compounds 2 and 8 revealed the former to be an ERK inhibitor, and the latter a BRAF inhibitor, reinforcing the notion that the pathway forms a central axis in the disease pathophysiology. Compound 2 (CAS #449732–51–8) is an analog of Ulixertinib and a potent ERK2, and likely ERK1, inhibitor^31^. Compound 8 is desmethyl-Dabrafenib, the desmethyl- metabolic byproduct of Dabrafenib that, in the human gut, is either excreted or reabsorbed into the bloodstream^32^. Given that Dabrafenib is a marketed product^33–35^, we therefore reasoned that compound 8 might likewise be able to be used in humans, whereas there are less data available for compound 2. For this reason, we focused the remainder of our work on compound 8, annotated henceforth as dmDf.

Finally, our data suggest that the LC50 (lethal concentration required to kill 50% of the population) of dmDf in zebrafish falls between 1 and 10 μM (Fig. 1). This concentration is some four orders of magnitude greater than the effective *in vivo* exposure (0.26 nM, Supplementary Fig. 4b) of dmDf that sufficiently rescues both mandibular and neurodevelopmental defects. These findings suggest that effective low-dosing of RAS/MAPK pathway inhibitors, such as dmDf, has the potential to safely ameliorate developmental KS phenotypes in human.

### Dabrafenib does not rescue a zebrafish model of KS

Among the numerous clinical trials involving RAS/MAPK signaling, small molecules targeting tumors that express *BRAF* mutations have been among the furthest to advance^36^. For example, Dabrafenib, a small molecule designed against the hyperactivated BRAF V600E mutant, has shown efficacy in adults with solid tumors, most notably malignant melanoma^33–35^. Given the apparent efficacy of dmDf in ameliorating KS in our zebrafish model, we wondered whether Dabrafenib might have a similar effect, which could accelerate its administration in KS patients. We treated *kmt2d* morphants with five serial dilutions of Dabrafenib, (1 nM to 1 µM), and we scored embryos for amelioration of CE defects at the 8–10 somite stage. Blind scoring of biological duplicates did not result in significant amelioration, with marginal improvements observed at the 50 nM and 100 nM doses (Supplementary Fig. 5). This result does not preclude the potential utility of Dabrafenib in the context of human metabolism or postnatal phenotypes. However, our results indicate that, compared with Dabrafenib, dmDf potentially shows greater therapeutic promise.

### dmDf attenuates hyperactive MEK signaling *in vivo*

**Figure 4:**
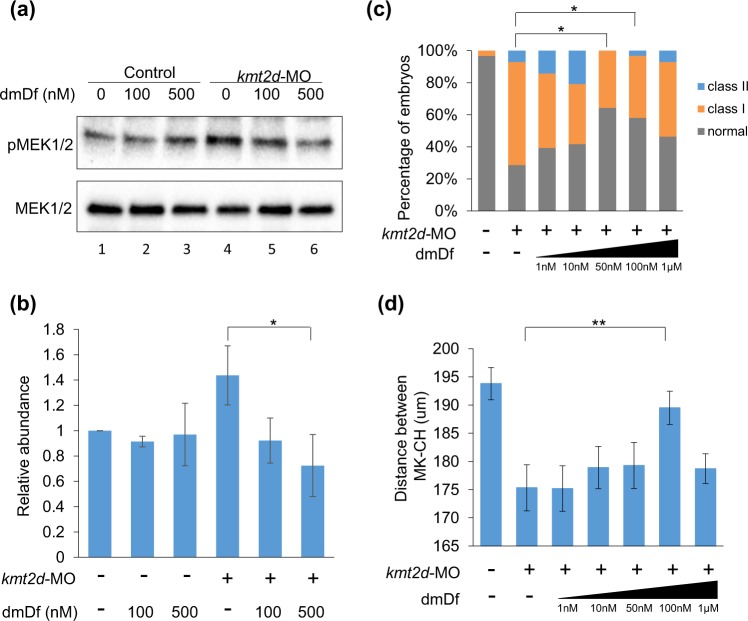
dmDf attenuates hyperactive MEK signaling *in vivo*. (**a**) Western blot analysis for the level of pMEK1/2 from the head lysate of 5 dpf zebrafish embryos. Depletion of *kmt2d* (lane 4) elevates the abundance of pMEK1/2 in comparison to control embryos (lane 1). Treatment with dmDf (100 nM and 500 nM, in lanes 5 and 6, respectively) attenuates hyperactivation of MEK1/2. Full-length blots are presented in Supplementary Fig. 3. (**b**) Summary of the relative levels of pMEK1/2 from three independent experiments. *p < 0.05 (n = 3). (**c**) *kmt2d* morphants were treated with serially increasing concentrations of dmDf. The embryos were scored for CE phenotypes as described earlier (n > 25 for each concentration. (**d**) The distance between MK and CH cartilages was measured from the same embryos as in (**c**). *p < 0.05; **p < 0.01. Error bars show SEM (standard error of the mean).

The phenotypic rescue of our zebrafish KS models by dmDf predicts a successful attenuation of hyperactive MEK signaling. To test this hypothesis directly, we suppressed *kmt2d*, harvested and pooled protein from embryo heads (n = 20/condition) and measured the abundance of phosphorylated (activated) MEK1/2 (pMEK1/2). Consistent with our morphological data, exposure of embryos to either 100 nM or 500 nM of dmDf restored the abundance of pMEK1/2 to levels indistinguishable from wild type embryos (Fig. 4a,b); neither concentration had an appreciable effect on pMEK1/2 abundance in uninjected embryos, an observation consistent with the lack of toxicity of the compound at these concentrations (Fig. 1b).

Finally, we carried out an assessment on the dose response of dmDf in our KS zebrafish model. Although it is technically difficult to study molecular interactions (such as with phospho-BRAF to report the amount of free BRAF at different drug concentrations) in the zebrafish system, dose-response data can provide information about maximal efficacy and potency. We treated *kmt2d* morphants with 1 nM to 1 µM of dmDf and we assessed the degree to which CE and jaw defects were rescued (Fig. 4c,d). We observed significant rescue of CE defects by 50 and 100 nM of dmDf, while only the 100 nM concentration of this compound was able to rescue the jaw layout (Fig. 4c,d). In both features, the ability to rescue increased with drug dosage up to 100 nM. Beyond that point, higher dosages did not improve efficacy any further.

### Potential utility of dmDf for KS-relevant neuroanatomical defects

Although the attenuation of CE and jaw defects by dmDf in our zebrafish models is encouraging, the clinical impact of the former phenotype is unclear. We therefore wondered whether we could use this compound to ameliorate phenotypes that, while still testable during zebrafish development, might be clinically relevant for young KS patients. Studies have shown that postnatal reversal of neurodevelopmental genetic insults can be efficacious in protecting against long-term neurocognitive defects if administered early in postnatal life^37^, possibly because neurological development in humans continues through childhood and adolescence^38^. Under this paradigm, we asked whether dmDf could restore neurogenesis in genetic KS zebrafish mutants.

Our prior and current genetic zebrafish models of KS were focused on the newly discovered *rap1a*/*rap1b* genes. However, from the perspective of clinical utility, dmDf should be efficacious in *kmt2d* mutants, since loss of function mutations of the human ortholog account for as much as 75% of KS^8–11^. Therefore, we supplemented our testing tools by creating a *kmt2d* CRISPR/Cas9 mutant. A guide RNA targeting exon 4 of *kmt2d* induced efficient (~99%) Cas9-mediated genome editing (Supplementary Fig. 6). Similar to *kmt2d* morphants and *rap1* CRISPR mutants, *kmt2d* CRISPR mosaic mutants (F0) exhibited defects in CE and jaw development; encouragingly, both phenotypes could be ameliorated significantly by administration of 100 nM of dmDf (Supplementary Fig. 7a–c).

**Figure 5:**
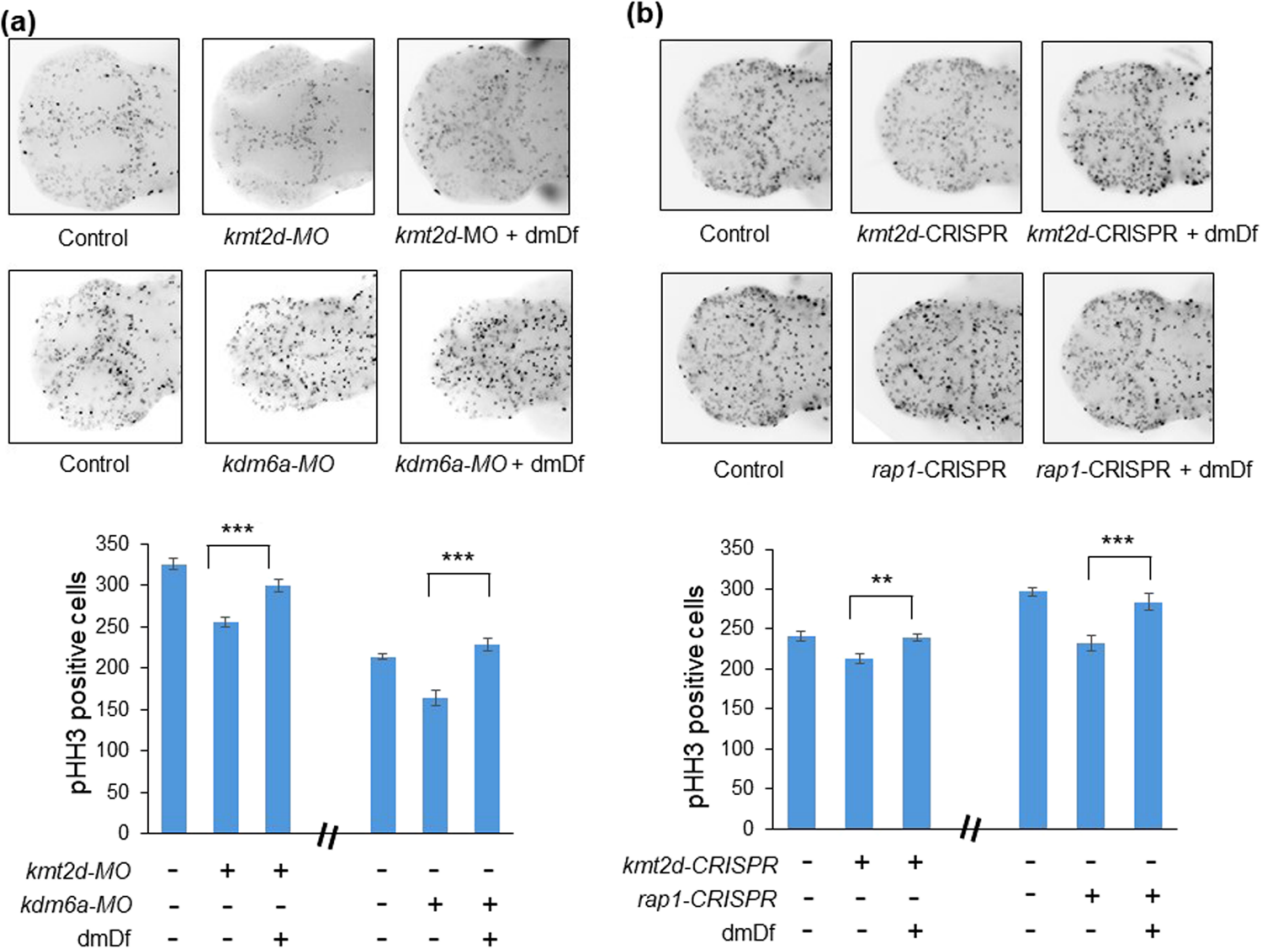
dmDf ameliorates cell proliferation defects in the brain caused by loss of kmt2d or rap1. (**a**) Depletion of *kmtd2* and *kdm6a* through morpholino injection leads to a significant reduction in the number of proliferating cells in the brain, visualized with immunostaining for phospho-histone H3 (pHH3) positive cells, in 2 dpf embryos. Treatment with dmDf significantly increases pHH3+ proliferating cells. (**b**) *kmt2d*-CRISPR and *rap1*-CRISPR embryos also exhibit a significant reduction in pHH3+ cells in the brain that can be rescued by treatment with dmDf. *p < 0.05; **p < 0.01; ***p < 0.001. Error bars show SEM (standard error of the mean).

Intellectual disability represents a major phenotype in KS, with some studies reporting almost 90% of patients to be affected^39^. Although the mechanistic basis of this phenotype is unknown and likely driven by multiple neurodevelopmental defects, we asked (1) whether our transient or stable KS zebrafish models exhibited quantitative neurodevelopmental phenotypes; and (2) whether any observed pathologies could be rescued by dmDf. Previous studies have shown that defects in proliferation in the developing zebrafish brain can be a proxy for neurodevelopmental and neurocognitive traits, especially in the context of microcephaly^40–43^, a phenotype reported commonly in KS^44^. As such, we quantified the total number of proliferating cells in the developing zebrafish brain by phospho-Histone H3 (pHH3) immunostaining at 2 dpf. Consistent with our predictions, *kmt2d* and *kdm6a* morphants, as well as *kmt2d-* and *rap1-* mosaic mutants exhibited a significant reduction of proliferating cells in the developing brain compared to sham-injected controls (Fig. 5). In each case, treatment with 100 nM dmDf restored the total number of proliferating cells to wild type levels (Fig. 5).

### dmDf rescues a stable *kmt2di mutant*

**Figure 6:**
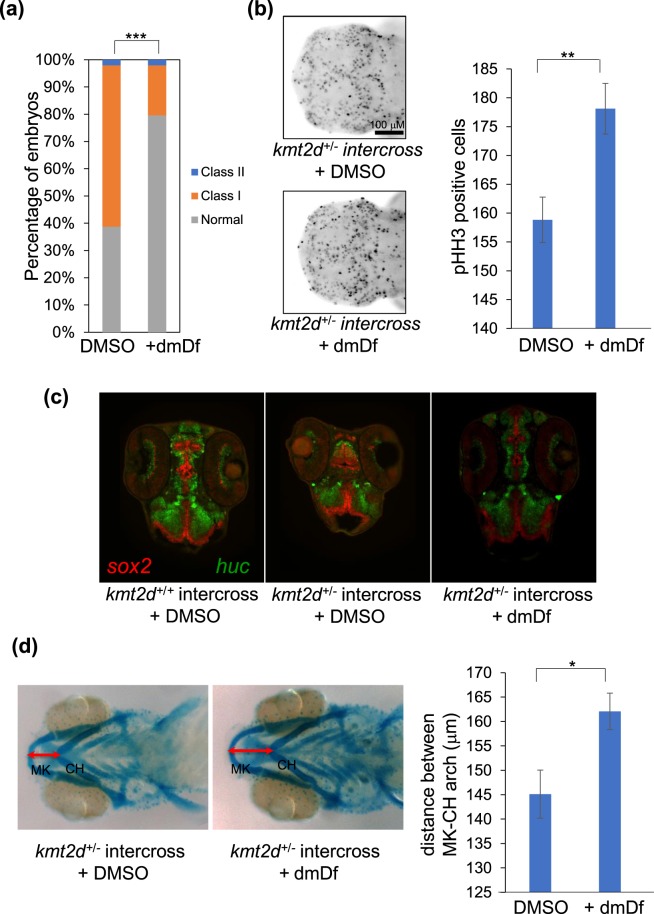
Treatment with dmDf rescues the defects of stable *kmt2d* mutant. *kmt2d*^+/−^ adults were in-crossed and the embryos were then collected at 1 dpf, 2 dpf and 5 dpf to assess dmDf efficacy. (**a**) dmDf ameliorates significantly the CE defect in *kmt2d*^+/−^ × *kmt2d*^+/−^ progeny. (**b**) Treatment with dmDf increases significantly the brain cell proliferation of *kmt2d*^+/−^ × *kmt2d*^+/−^ progeny. (**c**) administration of dmDf appears to ameliorate the differentiation delay in *kmt2d*^+/−^ × *kmt2d*^+/−^ progeny. (**d**) dmDf rescues significantly the jaw defect in *kmt2d*^+/−^ × *kmt2d*^+/−^ progeny. *p < 0.05; **p < 0.01; ***p < 0.001. Error bars show SEM (standard error of the mean).

Although our small molecule testing data in CRISPR F0 mosaic models are consistent with morphant phenotypes (and rescue), we recognize the necessity of testing the potential efficacy of dmDf in a stable genetic model that is a better genotypic proxy to human patients. Maintaining homozygous zebrafish CRISPR mutants for any KS gene is not possible due to embryonic lethality, a finding consistent with a mouse KS model^45^. However, we were able to maintain heterozygous *kmt2d* mutants bearing a 10 bp deletion in exon 6 (*kmt2d*^+/−^). This deletion induces a frameshift and premature termination that is predicted to lead to the translation of maximally a 191-residue peptide (instead of a full length 4967-amino acid long protein; Supplementary Fig. 8). Intercrossing *kmt2d*^+/−^ and genotyping progeny gave mendelian ratios at 1 dpf (26% wt, 47% −/+, 27% −/−) but showed skewing by 5 dpf (11% −/−), indicative of lethality. We therefore focused our phenotyping efforts in that time window, where we generated intercrosses from F1 progeny, exposed them either to sham or 100 mM dmDf and phenotyped them for CE (1 dpf), neurogenesis (2 dpf) and jaw development (5 dpf). For each phenotype, we observed significant amelioration (averaged measurements across entire zebrafish clutches, replicated). For CE, in contrast to 60% of embryos showing Class I or Class II defects, dmDf-treated clutches showed defects in only 20% of embryos (p < 0.001; Fig. 6a). Similarly, the F1 intercross progeny showed a significant decrease in the number of proliferating cells in the brain, that was likewise rescued by dmDf (Fig. 6b). Finally, given that previous studies have also highlighted neuronal differentiation defects in zebrafish KS model In addition to examining proliferation of cells in the brain, we assessed whether neural differentiation is affected^46^. To test this, we sectioned 5–10 affected animals at 2 dpf and marked undifferentiated-proliferative cells with sox2 (red in Fig. 6c) and differentiated/differentiating neural cells with huc (green in Fig. 6c). Consistent with the earlier work^46^, We found that the organization of both sox2+ and huc+ cells is affected, indicative of perturbed differentiation. However, administration of dmDf attenuated this pathology (Fig. 6c). Finally, we also saw rescue of the mandibular length at 5 dpf (Fig. 6d).

Taken together, these data support the transient MO and F0 rescue data with dmDf and bolster its candidacy as a therapeutic agent.

## Discussion

A persistent issue in the development of therapeutics for rare human genetic disorders is that the *ab initio* identification of small molecules of therapeutic value faces steep economic and regulatory barriers^47^. As such, the community is motivated to look for repurposing opportunities that are guided by biochemical knowledge of disease pathomechanism. We have shown previously that some of the pathognomonic defects of KS are likely driven by the hyperactivation of RAS/MAPK signaling, a pathway studied exhaustively in the context of neoplasia^22^. Here, we have taken advantage of this overlap to screen a focused set of diverse RAS/MAPK signaling antagonists as a means of providing additional support for a causal link between KS and RAS/MAPK signaling and to explore a possible route to therapeutics. Our screening progression path identified six hits covering all three tested RAS-MAPK signaling targets: BRAF, MEK, and ERK. We found that two compounds were able to repeatedly ameliorate KS-related defects in *kmt2d*, *rap1 and kdm6a* zebrafish models. Moreover, at 100 nM, a BRAF antagonist, desmethyl-Dabrafenib, is safe (to the extent that can be tested during zebrafish development) and efficacious at ameliorating all three tested KS-relevant pathologies: convergence and extension during gastrulation, jaw development, and cell proliferation in the developing zebrafish brain. The improvement for the neurological defects particularly draw our attention because neurological development in humans continues through childhood and adolescence^38^. Our study reveals the potential of dmDf for the restoration of neurological phenotypes in KS patients by its ability to facilitate brain cell proliferation. However, neurogenesis is a complicated process. A recent study of two KS zebrafish morphants (*kmt2d* and *kdm6a*), reported that loss of *kmt2d* and *kdm6a* leads to decreased neural cell differentiation^46^. Although not a perfect indication for a drug screen, we have also observed delayed differentiation in our *kmt2d* mutant; treatment with dmDf ameliorates this pathology. In addition, we also observed structural heart defects, a phenotype that has been also been reported previously in *kmt2d* and *kdm6a* morphants^46^. However, the poor dynamic range of this phenotype rendered it intractable to drug screening, while even if efficacious, the clinical application of any small molecule this early in human gestation is likely intractable; as such we focused on neurodevelopmental defects.

Retrospectively, the fact that two of 27 compounds showed robust evidence of *in vivo* rescue at a dose at least an order of magnitude below any observed toxicity supports our pathway-based hypothesis and the potential utility of BRAF or ERK inhibitors for KS. At the same time, it raises the question of why the success rate was not higher. This could be due to differences in absorption efficiency; immature metabolism of zebrafish embryos; or species-specific responses, including differences in target engagement. The fact that we could only detect 250 pM of our most efficacious compound in the exposure experiment (Supplementary Fig. 4b) suggests that zebrafish rescue assay conditions may not be conducive to high compound exposures. Nonetheless, given the lack of precedence for these kinds of experiments, we are encouraged by the discovery of two compounds, targeting different RAS-MAPK pathway members, that are able to rescue multiple phenotypes in independent zebrafish genetic models of Kabuki syndrome. We find the rescue of the stable *kmt2d* mutant most compelling, since it offers the most control for background uniformity.

Desmethyl-Dabrafenib is a metabolic byproduct of Dabrafenib, a currently prescribed anti-cancer agent, that is generated in the human gut and is either excreted or reabsorbed in the bloodstream, where it builds over time^32^. Given that the morphogenesis of the gut is not complete during our investigative window, the lack of efficacy of Dabrafenib is not surprising and might suggest that the metabolic derivative might be the key agent for rescue. Nonetheless, an abundance of caution is warranted. First, given that KS is a pediatric disorder, we do not know the tolerance of this population to these compounds. Second, there are fundamental dosing and toxicity questions that must be addressed when one transitions from treating acute somatic disorders to managing chronic germline conditions.

Finally, inasmuch as our data motivate us to pursue the utility of dmDf for KS, further, significant work will be required to assess how a rescue of early developmental phenotypes in zebrafish can translate to postnatal phenotypes in humans. Several recent studies have emerged that support this paradigm^48,49^. Nonetheless, experiments in additional preclinical species and detailed characterization of PK/PD in zebrafish embryos as well as mouse models are necessary. Such studies would enable us to test the efficacy of desmethyl-Dabrafenib and other RAS-MAPK pathway compounds when administered neonatally or postnatally; would facilitate the identification of suitable, potentially treatable phenotypes; and would help guide the establishment of clinical trial endpoints.

## Materials and Methods

All studies were conducted in accordance with the GSK Policy on the Care, Welfare and Treatment of Laboratory Animals and were reviewed and approved both by the Institutional Animal Care and Use Committee at GSK and by the Duke University Institutional Animal Care and Use Committee.

### Zebrafish embryo manipulation, microinjection and compound treatment

Morpholinos (MO) were obtained from Gene Tools (Gene Tools, LLC, Philomath, OR, USA) and have been described previously^15^; *kmt2d*-MO: 5′-AATCATTTATGTTTACTAACCTGCA-3′ (5 ng); *kdm6a*-MO: 5′-GGAAACGGACTTTAACTGACCTGTC (10 ng). *kmt2d*-MO and *kdm6a*-MO was injected into wild type (WT) Ekkwill (EK) zebrafish embryos obtained from natural matings at the 1–4 cell stage. CRISPR target sequences (*rap1a*: 5′-GTGTTGGGCTCTGGTGGTGT-3′ [120 pg], *rap1b*: 5′-TGCCAACACCTCCTGATCCG-3′ [120 pg]^15^, and *kmt2d*: 5′-GGGTGAGGTGCTGATAAACGTGG [50 pg]) were identified by crispr.mit.edu or CHOPCHOP (http://chopchop.cbu.uib.no/) and cloned into pT7-gRNA (Addgene, Cambridge, USA) following the protocol described in http://www.addgene.org/crispr/Chen/. Guide (g)RNAs were synthesized using the T7 MEGAshortscript kit (ThermoFisher Scientific). An injection solution of gRNA plus 100 pg Cas9 protein (PNA Bio) was injected into wild type Ekkwill (EK) zebrafish embryos obtained from natural matings at the 1-cell stage. Embryos were treated with compound at the 8-cell stage. All compounds were dissolved in dimethyl sulfoxide (DMSO; 10 mM), and subsequently diluted with water to 100 μM. Using egg water, we then diluted the 100 μM solution to the concentration specific to each experiment in egg water. Embryos were incubated at 28.5 °C and egg water containing compound was replaced daily until phenotyping, mass spectrometry, or immunoblotting analysis.

### Zebrafish embryo and larval phenotyping

For convergence and extension (CE) phenotyping, embryos were scored as described^27^ at the 8–10 somite stage, and images were captured using an AZ100 microscope and NIS Elements software (Nikon). To assess mandibular phenotypes, Alcian blue staining was used to stain cartilage structures in 5 dpf larvae as described^50^. Stained larvae were imaged in glycerol using an SMZ745T stereomicroscope (Nikon). The distance between Meckel’s (MK) and ceratohyal (CH) cartilages was measured on ventral bright field images with Image J (NIH). For brain cell proliferation assays, injected embryos were fixed in Dent’s solution at 2 dpf. Immunohistochemistry was performed with primary antibody for phospho-histone H3 (Santa Cruz Biotechnology; 1:500) and secondary antibody Alexa Fluor goat anti-rabbit IgG (ThermoFisher; 1:1000) according to standard procedures. Fluorescence signal on embryo heads was imaged with the Nikon AZ100 microscope and DS-Qi1MC digital camera using 200 μM Z-stacks to generate extended depth of focus images. Cell numbers were quantified using the ImageJ ICTN plugin (NIH). To assess the neural differentiation, 2 dpf embryos were fixed in 4% PFA overnight, followed by 30% sucrose incubation for another overnight. Embryos were then embedded in O.C.T. (Optimal Cutting Temperature) compound (Tissue-Trek) and sections were cut at 14 μm in a cryostat (LEICA CM3050S). The sections were then stained with sox2 (abcam) and huc (ThermoFisherScientific) antibodies for 1 hr. The images were captured using Nikon 90i microscope. Experiments were repeated at least twice with 30–40 embryos per condition (CE); 30–40 embryos for Alcian blue; and 20 embryos per condition (cell proliferation). Pairwise comparisons to vehicle (DMSO)-treated embryos for rescue efficacy were conducted using a χ^2^ test (CE) or Student’s t-test (mandible and cell proliferation).

### Validation of kmt2d-CRISPR reagents

To validate the genome-editing efficiency of *kmt2d*-CRISPR reagents, we harvested 10 gRNA/Cas9-injected embryos selected randomly at 1 dpf. Genomic DNA was extracted using 15 μl of 1X Taq buffer (New England Biolabs). The region targeted by *kmt2d* gRNA was PCR-amplified (Forward: 5′-AAGCAATGGCTATGGTTTGTTTA-3′ and Reverse: 5′-AAAGGAAGCTCTGTGCCTACC-3′). The PCR product was then denatured and re-annealed slowly (95 °C for 5 min, ramping down to 50 °C at 0.1 °C/sec, incubating at 50 °C for 10 min, and chilling to 4 °C at 1 °C/sec), and subjected to 15% polyacrylamide gel electrophoresis (PAGE) to detect the formation of heteroduplexes as described^51^. PCR products from six independent embryos were cloned into a pCR4-TOPO vector (Life Technologies), and Sanger sequenced to characterize the changes in the targeted region.

### Western blot analysis

Zebrafish larvae (5 dpf; n = 20) were decapitated and heads were homogenized with RIPA buffer (50 mM NaCl, 1% NP40, 50 mM Tris-HCl pH 7.5, 0.1% SDS, 0.5% Na deoxycholate, 1 mM Na_3_VO_4_, and 1 mM NaF). Total protein concentration was determined using the BCA Protein Assay Kit (Thermo Fisher Scientific) and 50 µg lysate per condition was subjected to 4–15% SDS-PAGE (Bio-Rad) and transferred to a PVDF membrane. Immunoblots were blocked in 3% BSA in PBS containing 0.1% Tween20 and probed with pMEK1/2 and MEK1/2 (#9121 and #9122, Cell Signaling Technology; 1:2000). Blots were developed using an enhanced chemiluminescence system, Super Signal West Pico Chemiluminescent Substrate (Thermo Fisher Scientific), visualized on a ChemiDoc (Bio-Rad) and quantified by Quantity One (Bio-Rad).

### Mass spectrometry to determine compound uptake in zebrafish embryos

WT EK embryos were transferred to a 12-well plate at the 8-cell stage and bathed in egg water containing 0.001% DMSO or 100 nM of each compound (n = 30/condition). Embryos were incubated at 28 °C; egg water containing compound was refreshed every 24 hours until harvest. Compound exposure was assessed essentially as described^30^; larvae were anaesthetized on ice, washed once with fresh compound-free ice-cold egg water, washed once with ice-cold PBS, and then transferred to a microfuge tube and centrifuged briefly before removing all excess liquid. Samples were macerated using a plastic pestle and frozen at −20 °C prior to analysis. Mass spectrometry analysis was conducted at Covance Laboratories (Cary, North Carolina, USA). Briefly, the macerated larvae were diluted in 1 ml water/acetonitrile (50/50), sonicated for 3 × 10 s and vortexed. For all compounds, a control sample containing embryos exposed to 0.001% DMSO was analyzed as a control. Calibration curves were generated by spiking aliquots of control zebrafish homogenate (prepared as described above) with known amounts of compound 29 (N3521-4-2).

### Data availability

The datasets generated during and/or analyzed during the current study are available from the corresponding author on request.

## Electronic supplementary material

Supplementary information
