# Supplementary material for: Small molecule inhibition of RAS/MAPK signaling ameliorates developmental pathologies of Kabuki Syndrome
Source: Sci Rep. 2018 Jul 17;8:10779. doi: 10.1038/s41598-018-28709-y (PMC6050262; doi:10.1038/s41598-018-28709-y)
Supplement: Supplementary file 1 — Supplementary information [file 41598_2018_28709_MOESM1_ESM.pdf]

# Supplementary Information

# Small molecule inhibition of RAS/MAPK signaling ameliorates developmental pathologies of Kabuki Syndrome

I-Chun Tsai[1,†], Kelly McKnight[1, †], Spencer U. McKinstry[1], Andrew T. Maynard[2], Perciliz L. Tan[1], Christelle Golzio[1], C. Thomas White[2], Daniel J. Price[3], Erica E. Davis[1], Heather Amrine-Madsen[2], Nicholas Katsanis[1,*]

[1] Center for Human Disease Modeling, Duke University School of Medicine, Durham, NC 27701

[2] Target Sciences, GlaxoSmithKline, Research Triangle Park, NC 27709

[3] Platform Technology and Science, GlaxoSmithKline, Research Triangle Park, NC 27709

[†]These authors contributed equally to this report

*Correspondence to NK: nicholas.katsanis@duke.edu

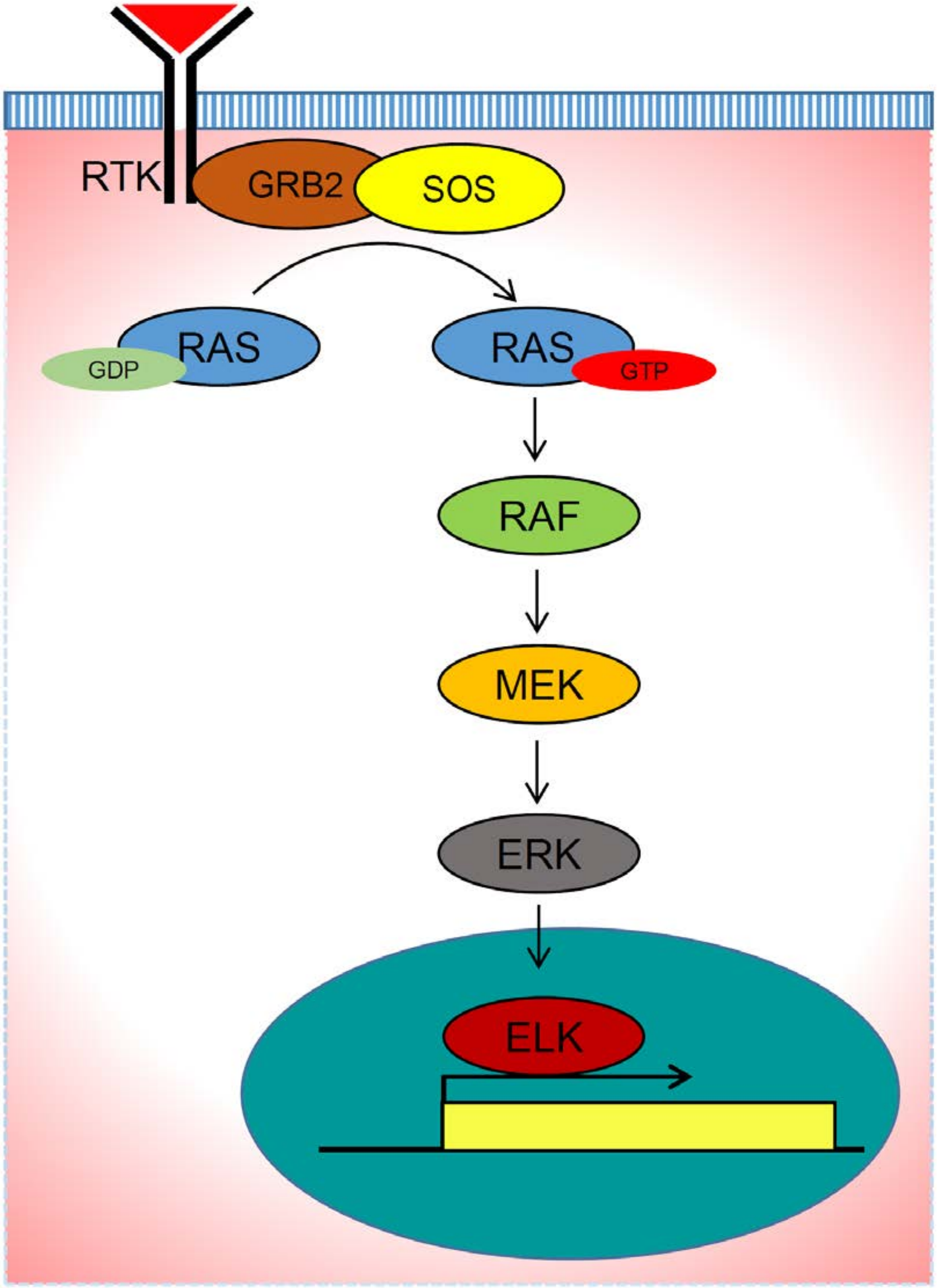


.

**Supplementary Figure 1: Ras/MAPK signaling pathway.** Schematic representation of the Ras/MAPK pathway.

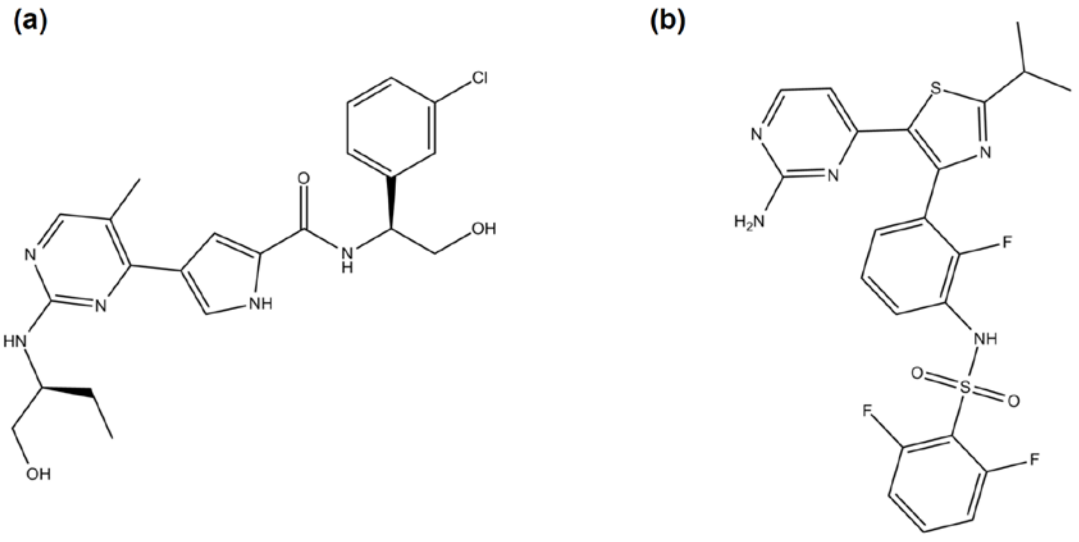


**Supplementary Figure 2: Chemical structure of compounds that rescued the CE and jaw defects in zebrafish KS models.** (a) Compound 2, an ERK inhibitor. (b) Compound 8, the BRAF inhibitor desmethyl-Dabrafenib.

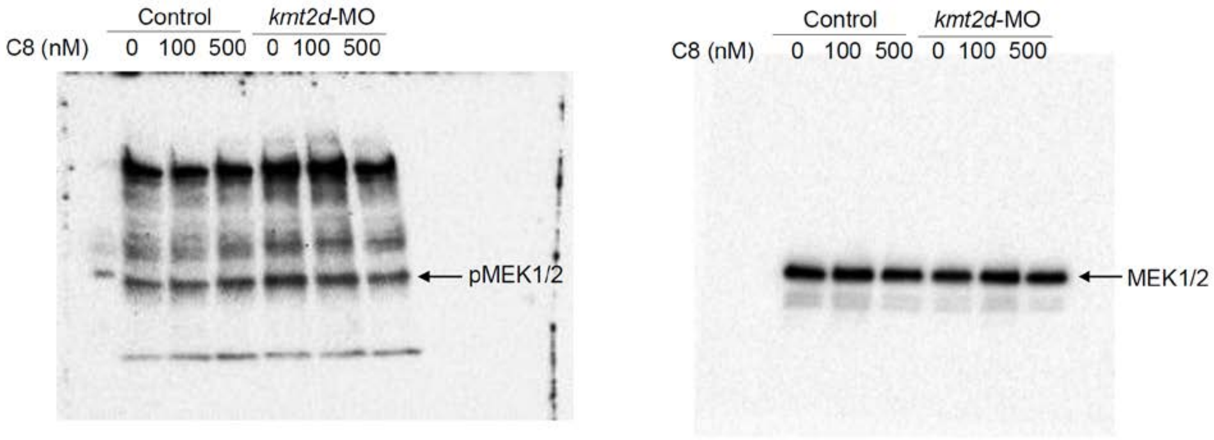


**Supplementary Figure 3: Full-length blots of Figure 5a.** Western blot analysis for the level of pMEK1/2 (left blot) and MEK1/2 (right blot) from the head lysate of 5 dpf zebrafish embryos.

(a)

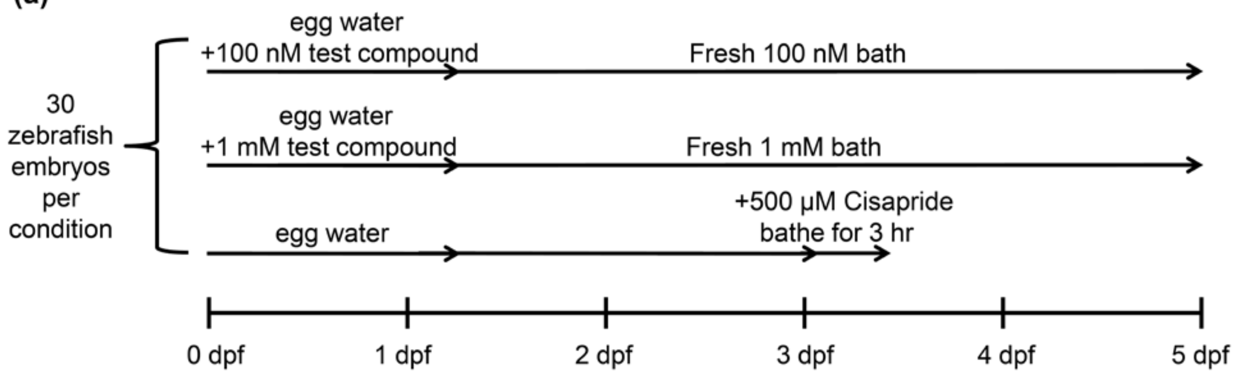


(b)

| Compound | MOA target | 100 nM dose | 1 µM dose |
|---|---|---|---|
| 8 | B-RAF | 0.26 nM | 4.45 nM |
| 2 | ERK1/2 | 0.05 nM | 3.27 nM |
| 29 | dead B-RAF analog | ** | ** |
| Cisapride | 5-$HT_4$ receptor agonist | 5.56 µM from 50 µM dose | 20.5 µM from 500 µM dose |
| 6 | B-RAF | 0.06 nM | 0.83 nM |
| 22 | ERK1/2 | 0.01 nM | 1.47 nM |

**Supplementary Figure 4: Successful delivery of small molecules into zebrafish embryos.** (a) Experimental design and sample preparation for the mass spectrometry (MS) analysis of compound concentration. (b) Quantitative MS analysis results of compound exposure in embryos. The right-most two columns give the concentrations of each compound detected by MS in zebrafish, quantifying *in vivo* exposures resulting from egg water compound doses of 100nM and 1µM.

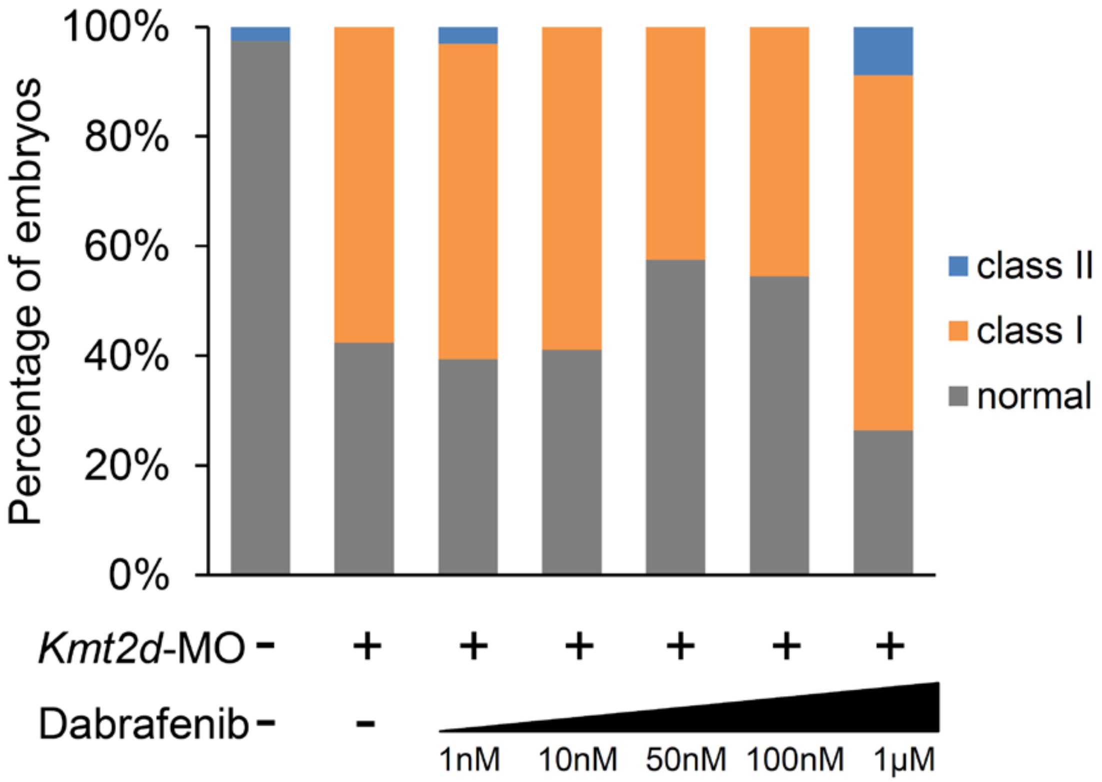


**Supplementary Figure 5: The efficacy of Dabrafenib, a BRAF inhibitor, to rescue KS defects.** *kmt2d* morphants were treated with serially increasing concentrations of Dabrafenib. The embryos were scored for CE phenotype as described earlier (n> 30 for each concentration).

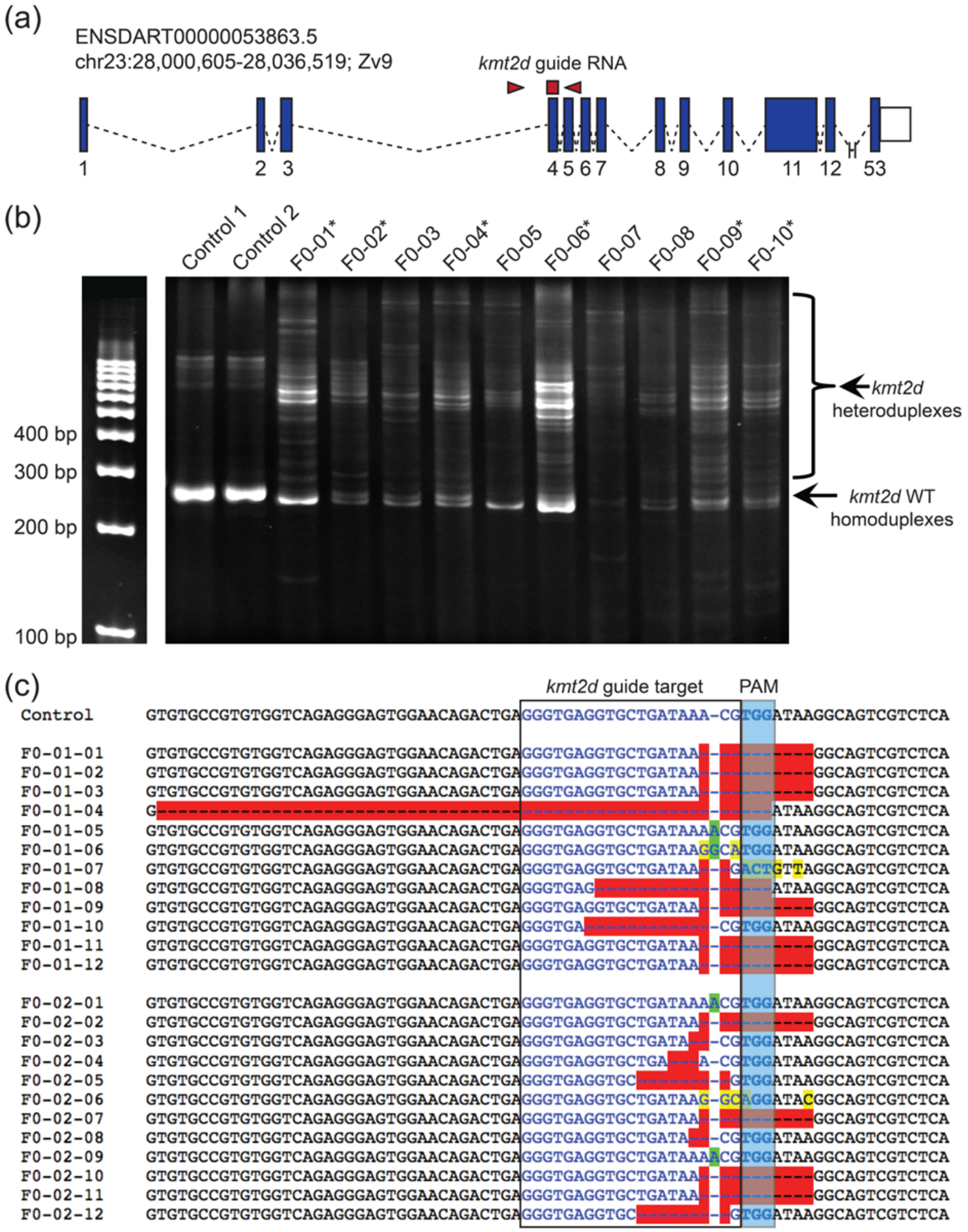


**Supplementary Figure 6: Validation of genome editing efficiency in *kmt2d*-CRISPR F0s.** (a) Schematic representation of the zebrafish *kmt2d* transcript. Blue boxes: exons; red box: gRNA targeted site; red arrowheads: primers for amplifying the potential mutation region. (b) Polyacrylamide gel electrophoresis (15% PAGE) of 2

control and 10 *kmt2d*-CRISPR F0 embryos shows the presence of heteroduplexes, indicating targeting events in CRISPR F0s. The PCR products of the embryos annotated with an asterisk (*) were then cloned into pCR4 for Sanger sequencing. (c) Representative Sanger sequencing results for an un-injected control embryo and 12 randomly selected clones of F0 embryos (F0-01 and F0-02) injected with *kmt2d* gRNA/Cas9 show insertion and or deletion events in the target region. The protospacer adjacent motif (PAM) sequence for gRNA is shown in blue box. Green box: insertion; red box: deletion; and yellow box: change.

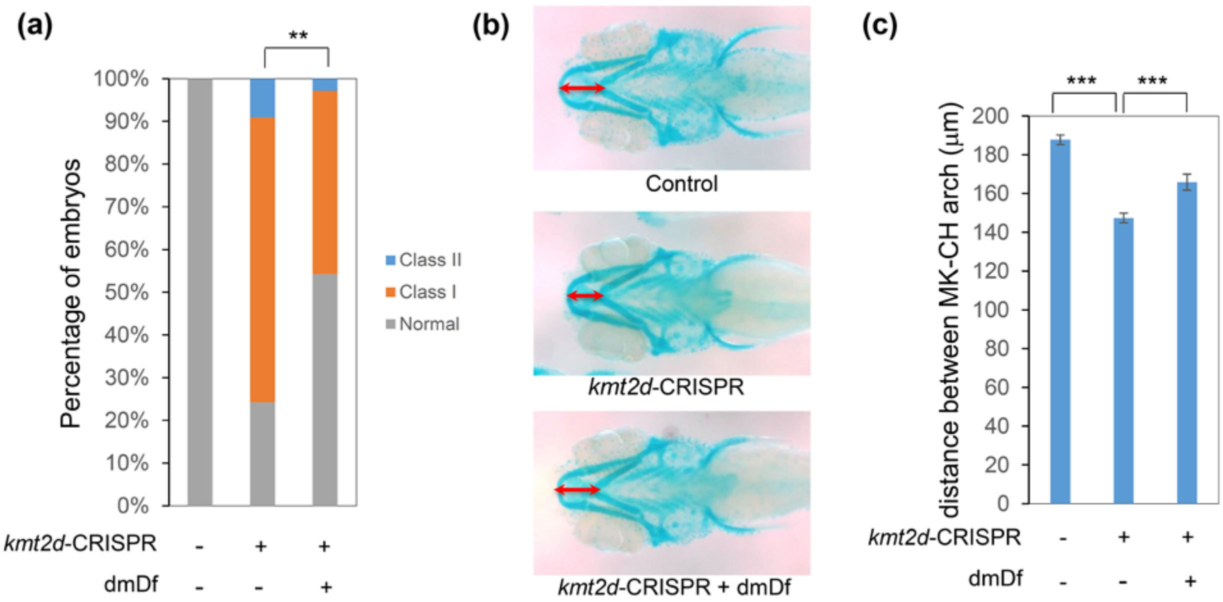


**Supplementary Figure 7: Treatment with dmDf rescues the developmental defects of *kmt2d*-CRISPR F0 mutants.** (a) Similar to *kmt2d*-MO, *kmt2d*-CRISPR F0s also exhibit CE defects, and treatment with dmDf ameliorates the CE defects in *kmt2d*-CRISPR F0 embryos. (b) 5 dpf embryos were stained with Alcian blue to visualize jaw layout. *kmt2d*-CRISPR F0s exhibit the same change in jaw layout as *kmt2d*-MO-injected embryos, with a reduced distance between MK and CH cartilages. Treatment with dmDf ameliorates the jaw defects seen in kmt2d-CRISPR F0 embryos. (c) Quantitative measurement of the distance between MK and CH cartilages. ***: p< 0.001. Error bars show SEM (standard error of the mean).

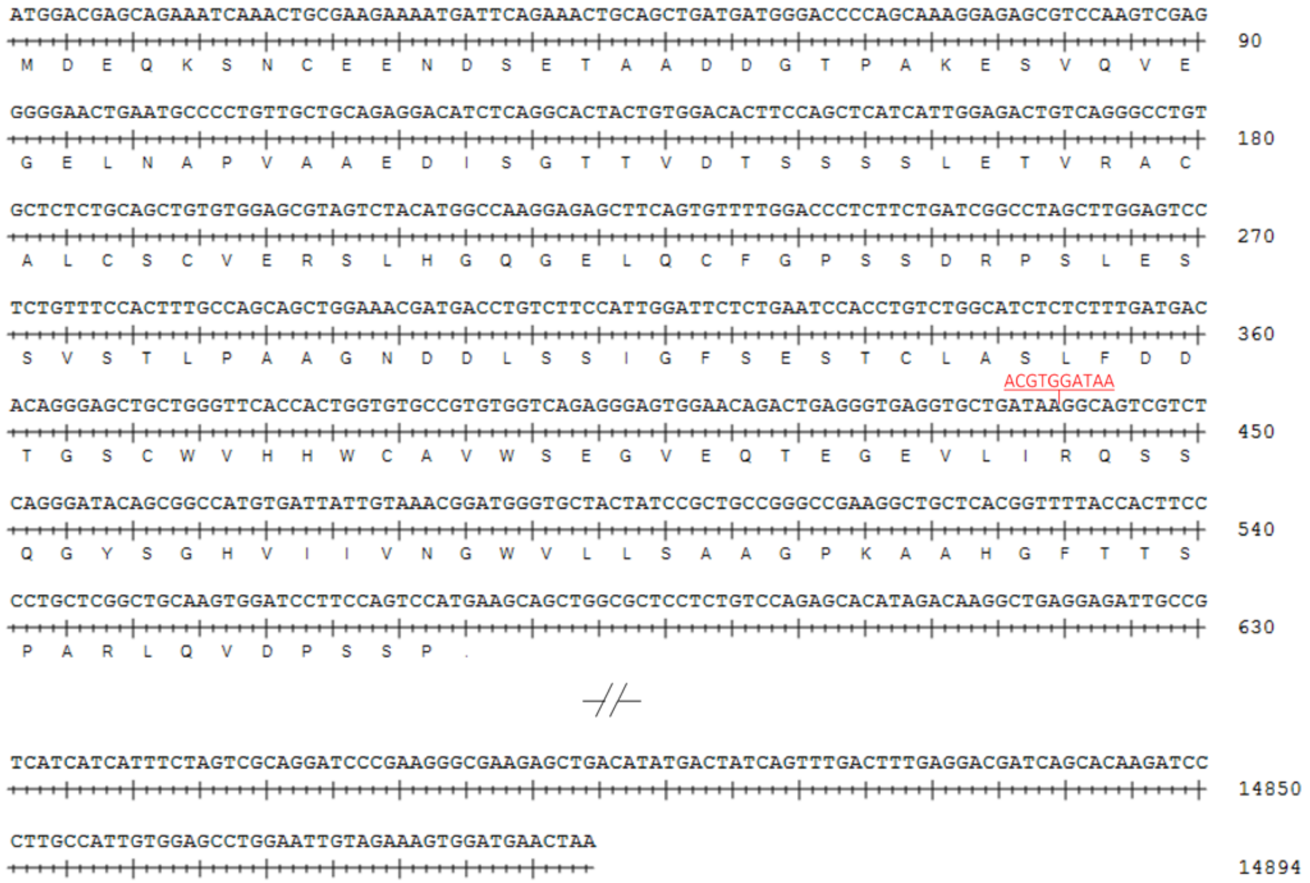


**Supplementary Figure 8: Mutation of stable *kmt2d*-CRISPR mutants.** *kmt2d* mutants carry a 10bp deletion in exon 6 (red DNA sequence). This deletion results in a frameshift and premature termination that is predicted to encode maximally a 191-residue peptide (instead of a full length 4967-amino acid long protein).
